# Microbiota-Derived Short-Chain Fatty Acids: New Road in Colorectal Cancer Therapy

**DOI:** 10.3390/pharmaceutics14112359

**Published:** 2022-11-01

**Authors:** Sara Gomes, Fátima Baltazar, Elisabete Silva, Ana Preto

**Affiliations:** 1 Centre of Molecular and Environmental Biology (CBMA), Department of Biology, Campus de Gualtar, University of Minho, 4710-057 Braga, Portugal; 2 Institute of Science and Innovation for Bio-Sustainability (IBS), University of Minho, 4710-054 Braga, Portugal; 3 Life and Health Sciences Research Institute (ICVS), School of Medicine, University of Minho, 4710-054 Braga, Portugal; 4 Department of Life Sciences, Brunel University (BU), London UB8 3PH, UK; 5 ICVS/3B’s PT Government Associate Laboratory, 4806-909 Braga/Guimarães, Portugal

**Keywords:** microbiota, short-chain fatty acids, acetate, butyrate, propionate, colorectal cancer

## Abstract

The colon microbiota is an important player in colorectal cancer (CRC) development, which is responsible for most of the cancer-related deaths worldwide. During carcinogenesis, the colon microbiota composition changes from a normobiosis profile to dysbiosis, interfering with the production of short-chain fatty acids (SCFAs). Each SCFA is known to play a role in several biological processes but, despite their reported individual effects, colon cells are exposed to these compounds simultaneously and the combined effect of SCFAs in colon cells is still unknown. Our aim was to explore the effects of SCFAs, alone or in combination, unveiling their biological impact on CRC cell phenotypes. We used a mathematical model for the prediction of the expected SCFA mixture effects and found that, when in mixture, SCFAs exhibit a concentration addition behavior. All SCFAs, alone or combined at the physiological proportions founded in the human colon, revealed to have a selective and anticancer effect by inhibiting colony formation and cell proliferation, increasing apoptosis, disturbing the energetic metabolism, inducing lysosomal membrane permeabilization, and decreasing cytosolic pH. We showed for the first time that SCFAs are specific towards colon cancer cells, showing promising therapeutic effects. These findings open a new road for the development of alternatives for CRC therapy based on the increase in SCFA levels through the modulation of the colon microbiota composition.

## 1. Introduction

According to the latest statistics from the World Health Organization, in 2020, colorectal cancer (CRC) was responsible for 1.93 million new cancer cases and approximately 930 600 cancer-related deaths worldwide [1]. These statistics highlight the magnitude of this health issue and the urgency in developing more specific and effective approaches to prevent and treat CRC.

Colonic dysbiosis has been identified as a key factor associated with CRC development. In this regard, the adoption of specific dietary patterns, such as the ingestion of dairy products, has been related to CRC prevention, since they contribute to the regulation of the intestinal microbiota composition [2,3,4,5]. Recently, the intestine microbiota was officially recognized as a hallmark of cancer, with a profound impact on protective or deleterious effects on cancer development, malignant progression, and response to therapy [6]. *Propionibacterium* ingested from dairy products is present in the intestinal microbiota, being one of the main producers of short-chain fatty acids (SCFAs), namely acetate, butyrate, and propionate [7].

Acetate, butyrate, and propionate are usually present in the colon and stool at physiological conditions in an approximate molar ratio of 60:15:25, respectively [8]. It has been demonstrated that specific changes in the human intestinal microbiota (dysbiosis) affect the amount and type of metabolites produced by colonic microbes. In this regard, a significant decrease in SCFA concentrations accompanied by an elevated colon pH has been reported in patients with CRC compared to healthy individuals [9,10,11].

There are some reports about the role of SCFAs in cancer cells, suggesting that they affect several biological processes [12]. The potential protective effects of butyrate against cancer cells are linked to the inhibition of cell differentiation, promotion of cell-cycle arrest and apoptosis, inhibition of the inflammatory response, and modulation of histone acetylation [13,14,15,16,17,18]. Propionate triggers antisurvival mechanisms such as apoptosis, necrosis, or autophagy, being also associated with the inhibition of inflammatory responses and modulation of histone acetylation [18,19,20]. Our group and others have demonstrated that acetate also presents biological effects in CRC cells, such as the inhibition of proliferation, induction of apoptosis, promotion of lysosomal membrane permeabilization with release of cathepsin D, and an alteration in the energetic metabolism through the modulation of monocarboxylate transporter expression [18,19,21,22,23].

While it is accepted that the colon of healthy individuals and CRC patients are exposed to different SCFAs in combination [8], to date, the majority of studies investigating their potential role in CRC prevention and treatment have been carried out on individual SCFAs. Only a limited number of studies have investigated the effects of combinations of SCFAs on biological processes in CRC cells, namely concerning their ability to modulate histone acetylation in human HT29 cells. However, there are several other processes in which they could play a role [24,25].

It is possible to study mixtures in toxicology through well-established mathematical models for the calculation of expected additivity and identification of additive, synergistic, or antagonistic effects [26,27,28]. However, to the best of our knowledge, this approach has never been applied to SCFA mixtures. The concentration addition model (CA) defines additivity by assuming that the mixture constituents have similar mechanisms of action, meaning that any component can be replaced partially or totally with another, without changing the overall mixture effect [29,30,31]. In addition, the effect of each individual component is in proportion to its concentration, contributing in this way to the global joint effect [28]. There are other predictive models available; however, since CA has been used to assess combination effects of agents with a common site of action, we believed that this was the most effective one for predicting the joint toxic effects of SCFAs.

Considering the relevance of a good understanding of the effects of SCFAs at physiological levels, in this work, we aimed to investigate the impact of mixtures of SCFAs on the biological phenotype of normal colon-derived NCM460 and CRC-derived RKO and HCT-15 cells.

## 2. Materials and Methods

### 2.1. Cell Lines and Culture Conditions

The work was carried out in three cells lines: one cell line derived from the normal colon, NCM460, and two cell lines derived from human colorectal cancer (CRC), RKO and HCT-15. NCM460 is a normal human colon mucosal epithelial cell line, and it was obtained from INCELL (San Antonio, TX, USA) [32]. These cells were grown in RPMI 1640 medium (with stable glutamine) (Biochrom, Berlin, Germany) supplemented with 10% (*v*/*v*) heat-inactivated fetal bovine serum (FBS; Biochrom, Berlin, Germany) and a mixture of penicillin–streptomycin at a final concentration of 1% (*v*/*v*) (5000 Units/mL of penicillin and 5000 μg/mL of streptomycin). The RKO cell line was first described by Michael Brattain and is derived from a primary colonic carcinoma [33]. These cells are microsatellite-unstable and harbor a BRAF mutation [34]. They were cultured in Dulbecco’s Modified Eagle’s Medium (DMEM; Biochrom, Berlin, Germany), supplemented with 10% (*v*/*v*) heat-inactivated fetal bovine serum (FBS; Biochrom, Berlin, Germany) and a mixture of penicillin–streptomycin at a final concentration of 1% (*v*/*v*) (5000 Units/mL of penicillin and 5000 μg/mL of streptomycin). HCT-15 cell line is derived from an adenocarcinoma specimen of human colon, removed during the normal course of a surgery [35,36]. They are microsatellite-unstable and harbor a KRAS mutation [34]. These cells were cultivated under the same conditions as the NCM460 cells. All cell lines were grown and maintained in 25 cm^2^ or 75 cm^2^ tissue-culture flasks at 37 °C, under a humidified atmosphere containing 5% CO_2_. When cells reached 80% to 100% of confluence, the culture medium was removed, and cells were washed with 1 × phosphate-buffered saline (PBS) and trypsinized (trypsin 0.05% (*v*/*v*) dissolved in phosphate-buffered saline–ethylenediamine tetra acetic acid (PBS-EDTA) (Gibco^®^, Waltham, MA, USA)) at 37 °C, in a 5% CO_2_ atmosphere. After 5 min, the trypsin was neutralized with the addition of fresh culture medium and the desired volume of cell suspension was transferred to a new 25 cm^2^ or 75 cm^2^ tissue-culture flask, together with the corresponding volume of culture medium. All cells were manipulated under aseptic conditions in a flow chamber.

### 2.2. Short-Chain Fatty Acids (SCFAs) Solutions

Sodium acetate, sodium butyrate, and sodium propionate were purchased from Sigma Aldrich. SCFA solutions were used as supplied and stock solutions were made up in deionized sterile water. Stock solutions were at least 20 times more concentrated than the highest concentration tested, in order to prevent media dilution. Subsequent dilutions were freshly prepared before each experiment. All solutions were stored at 4 °C.

### 2.3. Sulforhodamine B Assay

The cytotoxic effects of each SCFA were determined using the sulforhodamine B (SRB) assay, which allows cell density determination based on the measurement of cellular protein content [37]. Cells were seeded in 24-well plates at a density of 1.5 × 10^5^ cells per well (for NCM460) and 1.0 × 10^5^ cells per well (for RKO and HCT-15), in a volume of 500 μL of complete culture medium (supplemented with 10% (*v*/*v*) heat-inactivated fetal bovine serum (FBS; Biochrom, Berlin, Germany) and a mixture of penicillin–streptomycin at a final concentration of 1% (*v*/*v*) (5000 Units/mL of penicillin and 5000 μg/mL of streptomycin)). After 24 h, the media was gently aspirated, and the cells exposed to acetate, butyrate, propionate, or mixture solutions in fresh cell-culture medium. Each individual plate also included three replicates of negative controls (no test agents) and three replicates of positive controls (culture media containing 5% DMSO). Forty-eight hours after the treatment, cells were washed with 1 × PBS and fixed in methanol containing 1% acetic acid (*v*/*v*) at −20 °C for, at least, 90 min. Then, the fixing solution was carefully removed, and the plates were allowed to dry at room temperature (RT). When completely dried, plates were incubated with 0.5% (*w*/*v*) SRB dissolved in 1% acetic acid (*v*/*v*) at 37 °C, protected from light. Ninety minutes later, the SRB solution was removed, and the plates were washed with 1% acetic acid (*v*/*v*) in order to remove the excess of SRB. Plates were then left to air dry at RT. Finally, 1 mL of 10 mM Tris pH 10 was pipetted to solubilize the SRB, plates were carefully agitated, and 200 µL of the final solution were placed in a 96-well microplate where the absorbance was read at 540 nm on a Molecular Devices SpectraMax Plus 384 Microplate Reader (Molecular Devices, San Jose, CA, USA).

Statistical analysis was performed using GraphPad Prism version 8.4.3 for Windows (GraphPad Software, La Jolla, CA, USA, www.graphpad.com). To reduce interexperimental variability, data were scaled between 0% (negative controls) and 100% effect (positive controls). Results were graphically presented as percentage of inhibition of cell growth vs. concentration (mM).

All individual compounds were tested in at least three independent experiments, containing eight increasing concentrations of the test chemical in triplicate.

### 2.4. Mixture Testing

Two mixtures containing the same three SCFAs (acetate, butyrate, and propionate), but combined at different ratios, were tested. In mixture I, the compounds were combined at their IC_50_, meaning that they were present at concentrations that produced the same effect–equipotent concentrations. A master solution of mixture A with each SCFA at the concentrations present in Table 1 was prepared. In order to obtain a concentration–response relationship, the master mixture was serially diluted, maintaining the ratio between each constituent unchanged but testing a range of higher and lower concentrations, as per a fixed-mixture-ratio design described by Altenburger et al. and Backhaus et al. [38,39]. Mixture II was prepared in a similar way, but this time, the mixture components were combined at a ratio of 60 acetate: 15 butyrate: 25 propionate, mimicking the molar ratio usually founded in the human colon [40]. Similar to the first mixture, a serial dilution covering a wide range of concentrations was prepared.

### 2.5. Calculation of Predictive Mixture Effects

The overall effect of each of the two mixtures was predicted based on the complete concentration–response curves of the single agents applying the CA model, as described previously [31].

### 2.6. Regression Modeling and Statistical Analysis

Nonlinear regression analysis of all SCFAs, individually or in mixture, was performed using a best-fit approach [41]. The data obtained from the SRB assay (% inhibition of cell growth) were fitted to appropriate dosimetric models (Logit, Weibull, or General Logit I) using GraphPad Prism version 8.4.3 for Windows (GraphPad Software, La Jolla, CA, USA, www.graphpad.com). All of the nonlinear regression models describe sigmoidal concentration–response relationships. A suitable best-fit model was selected based on a statistical goodness-of-fit principle, after independently fitting each equation to the same data set (Table 1). All data provided by SRB assay are from at least three independent experiments, run in triplicate.

### 2.7. Colony-Formation Assay

RKO and HCT-15 cell lines were seeded in 6-well plates at a density of 500 cells/mL and 600 cells/mL, respectively. After adhering for 24 h, cells were treated with fresh complete medium as negative control, as well as with the IC_25_ and the IC_50_ of each SCFA (54.74 and 81.04 mM of acetate, 1.71 and 10.84 mM of butyrate, 17.58 and 32.25 mM of propionate for RKO and 42.98 and 89.52 mM of acetate, 1.45 and 4.57 mM of butyrate, and 9.92 and 22.70 mM of propionate for HCT-15). Additionally, the IC_25_ and the IC_50_ of mixture II were also tested, as well as the corresponding concentrations of each SCFA in that mixture (28.51 mM of Mix II containing 11.17 mM of acetate, 4.28 mM of butyrate, and 7.13 mM of propionate; 47.61 mM of Mix II containing 28.57 mM of acetate, 7.14 mM of butyrate, and 11.90 mM of propionate for RKO; 4.79 mM of Mix II containing 2.87 mM of acetate, 0.72 mM of butyrate, and 1.20 mM of propionate; 13.67 mM of Mix II containing 8.20 mM of acetate, 2.05 mM of butyrate, and 3.42 mM of propionate for HCT-15). After 48 h of treatment, the medium was replaced with fresh medium. Cells were then allowed to grow for 5–7 days (the medium was changed every 3 days). The colonies were washed with PBS and fixed for 30 min with 6% glutaraldehyde and 0.5% crystal violet. The number of colonies was counted using ImageJ Software (National Institutes of Health, Bethesda, MD, USA), and the percentage of colonies was normalized against the negative control.

### 2.8. Cell-Cycle Analysis

Cell cycle was evaluated through the measurement of the DNA content. RKO and HCT-15 cell lines were seeded in 12-well plates at a final density of 1 × 10^5^ cells/mL. After adhering for 24 h, cells were treated with fresh complete medium as negative control, as well as with the IC_50_ of each SCFA (81.04 mM of acetate, 10.84 mM of butyrate, 32.25 mM of propionate for RKO and 89.52 mM of acetate, 4.57 mM of butyrate, 22.70 mM of propionate for HCT-15). Additionally, the IC_50_ of mixture II was also tested, as well as the corresponding concentrations of each SCFA in that mixture (47.61 mM of Mix II containing 28.57 mM of acetate, 7.14 mM of butyrate, and 11.90 mM of propionate for RKO; 13.67 mM of Mix II containing 8.20 mM of acetate, 2.05 mM of butyrate, and 3.42 mM of propionate for HCT-15). After 24 h of treatment, cells were collected, resuspended in 500 μL PBS and incubated on ice for 15 min. An amount of 1.5 mL of 96% (*v*/*v*) cold ethanol was added and the cells were incubated for 15 min on ice. Then, cells were washed, resuspended in 500 μL of PBS, and incubated with 50 μL of RNase A solution (200 μg/mL in sodium citrate 1% (*w*/*v*)) at 37 °C for 15 min. An amount of 50 μL propidium iodide (PI) staining solution (0.5 mg/mL in sodium citrate 1% (*w*/*v*)) was then added and the cells were vortexed and incubated at room temperature for 30 min in the dark. PI mean fluorescence was analyzed by flow cytometry using PE-A channel.

### 2.9. Annexin V/PI Staining Assay

RKO and HCT-15 cell lines were seeded in 12-well plates at a density of 1 × 10^5^ cells/mL. After adhering for 24 h, cells were exposed to the following treatments for 48 h: the IC_50_ and the IC_75_ of each SCFA (81.04 and 116.60 mM of acetate, 10.84 and 44.16 mM of butyrate, 32.25 and 56.89 mM of propionate for RKO and 89.52 and 161.55 mM of acetate, 4.57 and 14.56 mM of butyrate, 22.70 and 50.70 mM of propionate for HCT-15). Additionally, the IC_50_ and the IC_75_ of mixture II were also tested, as well as the corresponding concentrations of each SCFA in that mixture (47.61 mM of Mix II containing 28.57 mM of acetate, 7.14 mM of butyrate, and 11.90 mM of propionate; 77.32 mM of Mix II containing 46.39 mM of acetate, 11.60 mM of butyrate, and 19.33 mM of propionate, for RKO; 13.67 mM of Mix II containing 8.20 mM of acetate, 2.05 mM of butyrate, and 3.42 mM of propionate; 39.97 mM of Mix II containing 23.98 mM of acetate, 6.00 mM of butyrate, and 9.99 mM of propionate, for HCT-15). As negative control, cells were incubated with fresh complete medium and positive controls were incubated with 0.2 μM of staurosporin. After 48 h, both floating and attached cells were collected and washed in PBS. Cells were then resuspended in 100 mL of binding buffer and incubated with 5 μL of Annexin V-FITC (Detection Kit—ab14085) and 5 μL of propidium iodide (50 μg/mL) for 15 min in the dark. To measure autofluorescence, cells were incubated with or without each probe, separately. Samples were analyzed by flow cytometry. The monoparametric detection of PI fluorescence was performed using ECD-A channel and the monoparametric detection of Annexin V fluorescence performed using FITC-A channel.

### 2.10. Lactate-Production Assay

1 × 10^5^ cells/mL of RKO and HCT-15 cell lines were seeded in 24-well plates (one plate for each timepoint—12, 24, and 48 h). After adhering for 24 h, cells were incubated with fresh complete medium (untreated control) or exposed to the IC_50_ of each SCFA (81.04 mM of acetate, 10.84 mM of butyrate, 32.25 mM of propionate for RKO and 89.52 mM of acetate, 4.57 mM of butyrate, 22.70 mM of propionate for HCT-15). Additionally, the IC_50_ of the mixture II was also tested, as well as the corresponding concentrations of each SCFA in that mixture (47.61 mM of Mix II containing 28.57 mM of acetate, 7.14 mM of butyrate, and 11.90 mM of propionate for RKO; 13.67 mM of Mix II containing 8.20 mM of acetate, 2.05 mM of butyrate, and 3.42 mM of propionate for HCT-15). After the corresponding timepoint, 2 μL of the culture medium from each condition was collected to a 96-well plate. Then, 100 μL of the Lactate LO-POD enzymatic colorimetric kit from Spinreact (Girona, Spain) was added to each well. The plate was maintained light-protected for 15 min and the absorbance was read at 502 nm in Molecular Devices SpectraMax Plus 384 Microplate Read (Molecular Devices, San Jose, CA, USA). Statistical analysis was performed using GraphPad Prism version 8.4.3 for Windows (GraphPad Software, La Jolla, CA, USA, www.graphpad.com).

The 24-well plates containing the adherent cells were used for an SRB assay (according to the protocol described before) and the total biomass present in each condition was used for calculating the lactate production. Importantly, a standard lactate curve (30, 20, 10, 5, and 2 mM) was used to assess the lactate content of each sample, being the validity of the results assumed for an r^2^ > 0.98 of the standard curve.

### 2.11. Intracellular pH Evaluation

Variations on the intracellular pH were evaluated with the pH-sensitive probe BCECF-AM. RKO and HCT-15 cell lines were seeded in 12-well plates at a density of 1 × 10^5^ cells/mL. After adhering for 24 h, cells were exposed to the following treatments: IC_75_ of each SCFA (116.60 mM of acetate, 44.16 mM of butyrate, 56.89 mM of propionate for RKO and 161.55 mM of acetate, 14.56 mM of butyrate, 50.70 mM of propionate for HCT-15). Additionally, IC_75_ of the mixture II was also tested, as well as the corresponding concentrations of each SCFA in that mixture (77.32 mM of Mix II containing 46.39 mM of acetate, 11.60 mM of butyrate, and 19.33 mM of propionate, for RKO; 39.97 mM of Mix II containing 23.98 mM of acetate, 6.00 mM of butyrate, and 9.99 mM of propionate, for HCT-15). As negative controls, cells were incubated with fresh complete medium. After 48 h of treatment, both floating and attached cells were collected, washed, and resuspended in Hank’s balanced salt solution (HBSS). Cells were then stained with 1 μM of BCECF-AM for 30 min at 37 °C. Samples were analyzed by flow cytometry, with BCECF fluorescence mean detection being performed using FITC-A and PE-A channels. The percentage of cells exhibiting intracellular acidification was estimated from the percentage of cells displaying an FITC-A/PE-A ratio lower than control cells.

### 2.12. Lysosome Membrane Permeabilization Assessment

Lysosome membrane permeabilization (LMP) was assessed using Acridine Orange (AO) staining by flow cytometry. RKO and HCT-15 cell lines were seeded in 12-well plates at a density of 1 × 10^5^ cells/mL. After adhering for 24 h, cells were exposed to the following treatments: IC_75_ of each SCFA (116.60 mM of acetate, 44.16 mM of butyrate, 56.89 mM of propionate for RKO and 161.55 mM of acetate, 14.56 mM of butyrate, 50.70 mM of propionate for HCT-15). Additionally, IC_75_ of the mixture II was also tested, as well as the corresponding concentrations of each SCFA in that mixture (77.32 mM of Mix II containing 46.39 mM of acetate, 11.60 mM of butyrate, and 19.33 mM of propionate, for RKO; 39.97 mM of Mix II containing 23.98 mM of acetate, 6.00 mM of butyrate, and 9.99 mM of propionate, for HCT-15). As negative controls, cells were incubated with fresh complete medium. After 48 h, both floating and attached cells were collected, washed with PBS, and resuspended in PBS. Cells were then incubated with 1 μM AO for 15 min at 37 °C. Samples were analyzed by flow cytometry, with AO fluorescence detection being performed using PC5.5 channel.

## 3. Results

### 3.1. Short-Chain Fatty Acids Inhibit Cell Growth in a Dose-Dependent Manner with Higher Specificity to Colorectal Cancer Cells

The first main aim of this work was to evaluate the potential interactions between the three SCFAs (acetate, butyrate, and propionate) and assess whether these interactions could be accurately predicted using the mathematical concentration addition (CA) model. In order to have the data required for the mathematical predictions of mixture effects, extensive concentration–response analyses of all the single SCFA had to be performed [28].

**Figure 1:**
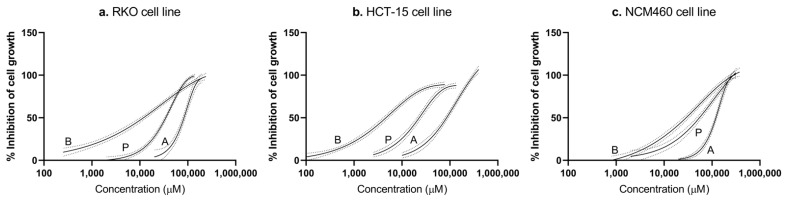
Regression models for the cytotoxic effects of all three mixture components in RKO (**a**), HCT-15 (**b**), and NCM460 (**c**) cells. The solid lines represent the regression models for acetate, butyrate, and propionate obtained in the SRB assay, following 48 h incubations. The labels are as follows: A—acetate, B—butyrate, P—propionate (for values see Table 2). Black dashed lines represent the upper and lower 95% CI for the regression fit. Experimental data were obtained from a minimum of three independent experiments run in triplicate.

The analysis of SRB results from each individual SCFA showed that all tested single agents inhibit cellular growth in a dose-dependent manner, meaning a decrease in cell number was observed with the increase in acetate, butyrate, and propionate concentrations (Figure 1). All SCFAs produced complete curves of percentages of inhibition of cell growth vs. drug concentration, both in CRC-derived cells (RKO and HCT-15) as in nonneoplastic colon cells (NCM460).

According to the nine dose-response curves obtained, the half-maximal inhibitory concentration (IC_50_) of acetate, butyrate, and propionate was calculated from the mean values of SRB inhibition of cell number after 48 h of treatment (81.04 mM of acetate, 10.84 mM of butyrate, 32.25 mM of propionate for RKO cell line; 89.52 mM of acetate, 4.57 mM of butyrate, 22.70 mM of propionate for HCT-15 cell line; and 117.30 mM of acetate, 25.96 mM of butyrate, 51.21 mM of propionate for NCM460 cell line) (Table 2). Additionally, the calculation of the selectivity index (SI), based on the IC_50_ values obtained for each SCFA in CRC cells in comparison with the values obtained for normal cells, allowed us to conclude that CRC cells are more sensitive to individual SCFAs than the nonneoplastic colon cells (SI > 1); thus, SCFAs are more selective to CRC cells [42].

### 3.2. Short-Chain Fatty Acid Mixtures Exhibit an Addictive Profile in Colorectal Cancer Cells

Two different mixtures were produced in which SCFAs were present at different mixture ratios. Mixture I was designed to assess the validity of the CA predictive model and contained the compounds combined at their IC_50_, meaning that they were present at equieffective concentrations. In turn, mixture II contained the compounds combined at the ratio of 60 acetate: 15 butyrate: 25 propionate, mimicking the molar ratio usually found in the human colon [40]. Seeing as mixture II contained the different SCFAs at proportions representative of a real-life scenario, this mixture was subsequently used for mechanistic studies.

**Figure 2:**
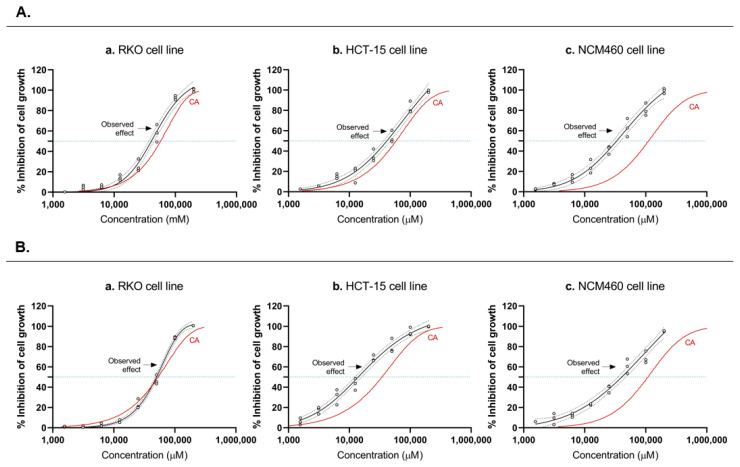
Predicted and observed effects of the SCFAs mixtures. (I,II) Predicted and observed effects of the SCFAs mixtures I (**A)** and II (**B**) of the three tested SCFAs in RKO (**Aa**,**Ba**), HCT-15 (**Ab**,**Bb**) and NCM460 (**Ac**,**Bc**) cells by SRB assay. Individual data points are represented by black circles, and the best-fit regression model is shown by the black line, labeled “observed effect”. Black dashed lines represent the upper and lower 95% CI for the regression fit. The solid red line shows the prediction according to concentration addition (CA). The blue dotted lines show the IC_50_ for each response curve (for values see Table 2). Experimental data were obtained from a minimum of three independent experiments run in triplicate.

Based on the concentration–response relationships of the individual chemicals, the concept of CA was used to mathematically predict the additive joint effects of the three drugs in both CRC-derived cells and nonneoplastic colon cells. Then, the combined effects of both mixtures (I and II) were tested experimentally, and the obtained curves (CA curve vs. experimental curve) were compared (Figure 2A,B). Data revealed low variability and led to a complete concentration–effect curve. For both CRC cell lines, RKO and HCT-15, the additive expectations according to CA agreed well with the experimental observations, suggesting that acetate, butyrate, and propionate, in both mixtures, exert a concentration-additive behavior. In the NCM460 cell line, the same conclusion cannot be assumed, since the CA curve is found slightly to the left of the observed effects. The results demonstrated that the additive effect seems to be generally followed, however it varies slightly between cell types, the effects being more evident in the CRC-derived cells, when compared with the noncancerous cell line. It is also possible to observe that butyrate has a more significant effect in the mixture than the other SCFA.

### 3.3. Short-Chain Fatty Acids Inhibit Colony Formation and Proliferation in Colorectal Cancer Cells

**Figure 3:**
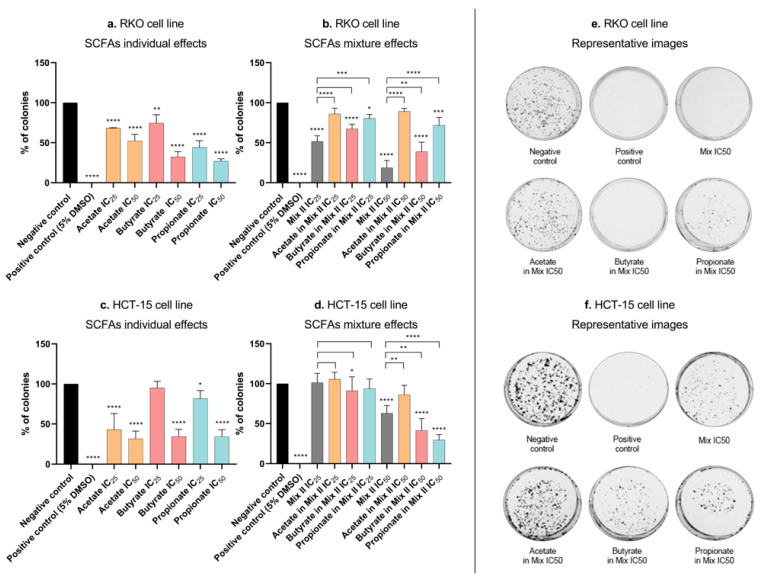
Colony formation effects induced by SCFAs, individually or in mixture, in CRC cells. (**a**–**d**) Cell-survival analysis by CFA assay in CRC cells treated with each SCFA, individually or in mixture, for 48 h. (**a**) RKO cells were incubated with the IC_25_ and the IC_50_ of each SCFA (54.74 and 81.04 mM of acetate, 1.71 and 10.84 mM of butyrate, 17.58 and 32.25 mM of propionate). (**b**) RKO cells were incubated with the IC_25_ and the IC_50_ of the mixture II, as well as the correspondent concentrations of each SCFA in that mixture (28.51 mM of Mix II containing 11.17 mM of acetate, 4.28 mM of butyrate, and 7.13 mM of propionate; 47.61 mM of Mix II containing 28.57 mM of acetate, 7.14 mM of butyrate, and 11.90 mM of propionate). (**c**) HCT-15 cells were incubated with the IC_25_ and the IC_50_ of each SCFA (42.98 and 89.52 mM of acetate, 1.45 and 4.57 mM of butyrate, 9.92 and 22.70 mM of propionate). (**d**) HCT-15 cells were incubated with the IC_25_ and the IC_50_ of the mixture II, as well as the correspondent concentrations of each SCFA in that mixture (4.79 mM of Mix II containing 2.87 mM of acetate, 0.72 mM of butyrate, and 1.20 mM of propionate; 13.67 mM of Mix II containing 8.20 mM of acetate, 2.05 mM of butyrate, and 3.42 mM of propionate). Data are from at least three independent experiments run in duplicate. Statistical analysis was performed using One-Way ANOVA (*p*-value 0.0332 (*), 0.0021 (**), 0.0002 (***) and <0.0001 (****)). (**e**,**f**) For both cell lines, representative images of the CFA of the IC_50_ of the mixture II as well as the correspondent concentrations of each SCFA in that mixture are presented. *p* > 0.001 compared with negative control cells.

**Figure 4:**
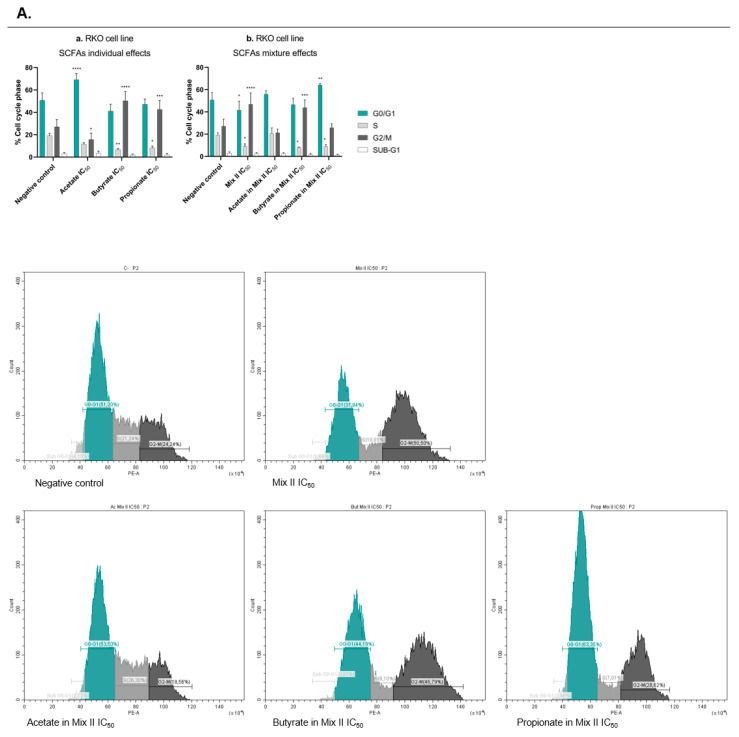

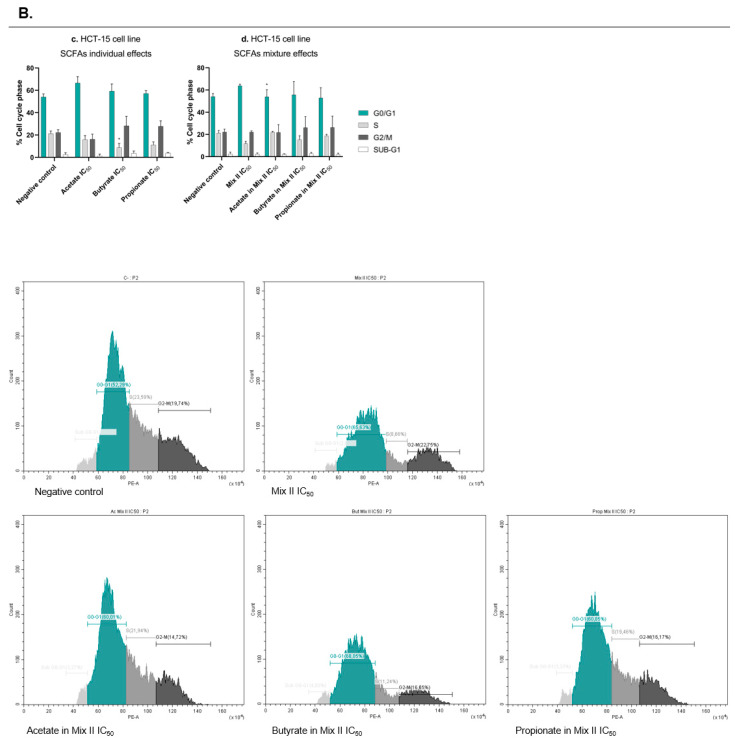
Cell-cycle effects induced by SCFAs, individually or in mixture, in CRC cells. Cell-cycle analysis by propidium-iodide staining by flow cytometry in RKO (**A**) and HCT-15 (**B**) cells treated with each SCFA, individually or in mixture, for 24 h. (**a**) RKO cells were incubated with the IC_50_ of each SCFA (81.04 mM of acetate, 10.84 mM of butyrate, 32.25 mM of propionate). (**b**) RKO cells were incubated with the IC_50_ of the mixture II, as well as the correspondent concentrations of each SCFA in that mixture (47.61 mM of Mix II containing 28.57 mM of acetate, 7.14 mM of butyrate, and 11.90 mM of propionate). (**c**) HCT-15 cells were incubated with the IC_50_ of each SCFA (89.52 mM of acetate, 4.57 mM of butyrate, 22.70 mM of propionate). (**d**) HCT-15 cells were incubated with the IC_50_ of the mixture II, as well as the correspondent concentrations of each SCFA in that mixture (13.67 mM of Mix II containing 8.20 mM of acetate, 2.05 mM of butyrate, and 3.42 mM of propionate). Data are from at least three independent experiments. Statistical analysis was performed using Two-Way ANOVA (*p*-value 0.0332 (*), 0.0021 (**), 0.0002 (***) and <0.0001 (****)). Representative histograms are represented. *p* > 0.001 compared with negative control cells.

The decrease in cell growth induced by each SCFA in the SRB assay might be due to decreased cell proliferation, increased cell death, or both. The next step of the work aimed at evaluating whether SCFAs were able to affect CRC cell survival and proliferation. It was also of our interest to compare the effects induced by mixture II with the effects induced by each SCFA individually at the concentrations presented in that mixture. This allowed us to understand if any of the SCFAs had more influence in the overall mixture effect. In this regard, cell survival was assessed through the measurement of the ability of single cells to grow into colonies after the treatment with each SCFA alone (at their IC_25_ and IC_50_), with mixture II (IC_25_ and IC_50_), and with each SCFA alone at a concentration present in the IC_25_ and IC_50_ of mixture II (for values, see Table 3). The results showed that, individually, each SCFA affected the CRC cells’ survival, with effects increasing proportionally to increasing concentrations (Figure 3a,c). Similarly, when cells were treated with mixture II, in RKO cells, the mixture seemed to follow mostly the effects from butyrate (Figure 3b,d). Afterwards, we performed a cell-cycle analysis in order to understand if SCFAs affect cell proliferation by interfering with the CRC cell cycle. We evaluated the effects of the treatments with each SCFA alone (IC_50_), with mixture II (IC_50_), and with each SCFA alone at a concentration presented in the IC_50_ of the mixture II. Cell-cycle analysis by flow cytometry was performed 24 h after incubation with the treatments, since we consider this to be the time required for cells to divide and, consequently, the best timepoint to evaluate effects on the cell cycle. The results showed that each single SCFA significantly affected the RKO cell cycle, with no significant effects in the HCT-15 cells (Figure 4a,c). Acetate induced cell-cycle arrest at the G0/G1 phase, while butyrate and propionate induced cell-cycle arrest at the G2/M phase. Considering the SCFA combined effects, in RKO cells, mixture II induced cell-cycle arrest in the G2/M phase, following the effects of butyrate and propionate (Figure 4b,d).

### 3.4. Short-Chain Fatty Acids Induce Apoptosis in CRC Cells

**Figure 5:**
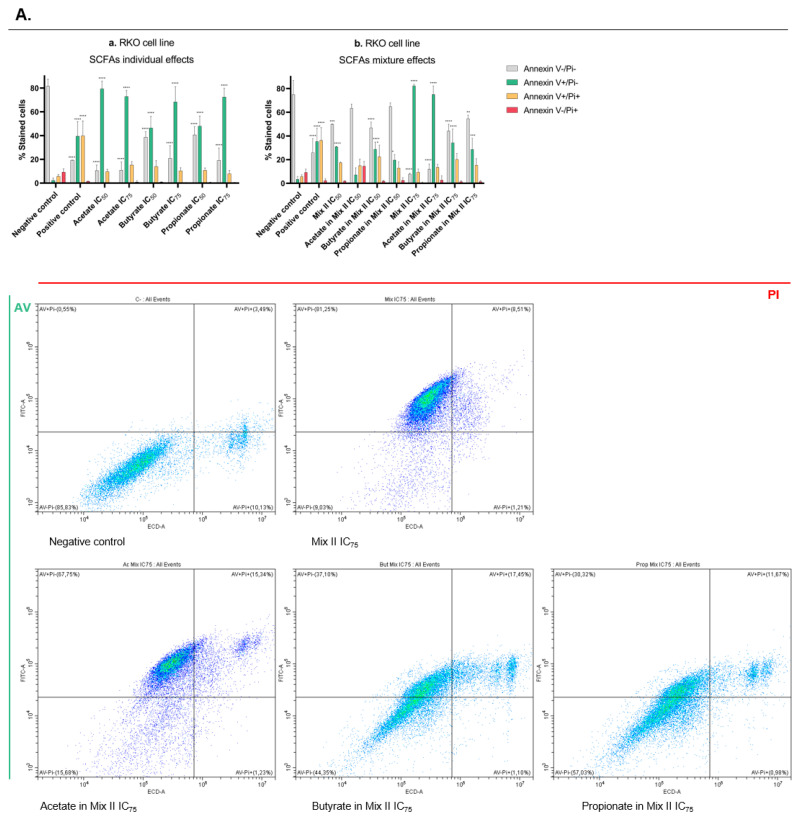

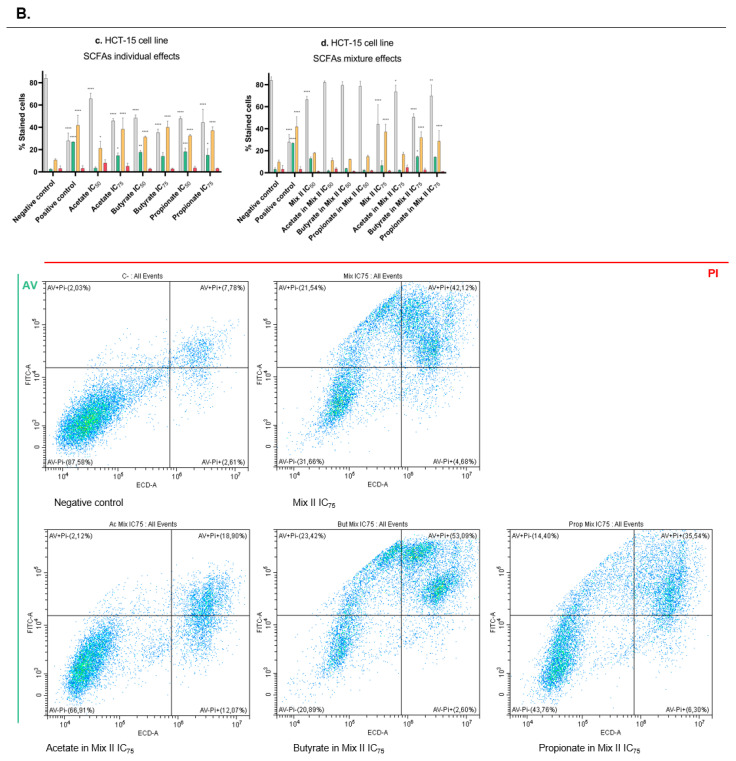
Apoptotic cell-death effects induced by SCFAs, individually or in mixture, in RKO (**A**) and HCT-15 (**B**) cells. (**a**–**d**) Cell-survival analysis by CFA assay in CRC cells treated with each SCFA, individually or in mixture, for 48 h. (**a**) RKO cells were incubated with the IC_50_ and the IC_75_ of each SCFA (81.04 and 116.60 mM of acetate, 10.84 and 44.16 mM of butyrate, 32.25 and 56.89 mM of propionate). (**b**) RKO cells were incubated with the IC_50_ and the IC_75_ of the mixture II, as well as the correspondent concentrations of each SCFA in that mixture (47.61 mM of Mix II containing 28.57 mM of acetate, 7.14 mM of butyrate, and 11.90 mM of propionate; 77.32 mM of Mix II containing 46.39 mM of acetate, 11.60 mM of butyrate, and 19.33 mM of propionate). (**c**) HCT-15 cells were incubated with the IC_50_ and the IC_75_ of each SCFA (89.52 and 161.55 mM of acetate, 4.57 and 14.56 mM of butyrate, 22.70 and 50.70 mM of propionate). (**d**) HCT-15 cells were incubated with the IC_50_ and the IC_75_ of the mixture II, as well as the correspondent concentrations of each SCFA in that mixture (13.67 mM of Mix II containing 8.20 mM of acetate, 2.05 mM of butyrate, and 3.42 mM of propionate; 39.97 mM of Mix II containing 23.98 mM of acetate, 6.00 mM of butyrate, and 9.99 mM of propionate). Cells were incubated with fresh complete medium as negative control and with 0.2 μM of staurosporin as positive control. Data are from at least three independent experiments. Statistical analysis was performed using Two-Way ANOVA (*p*-value 0.0332 (*), 0.0021 (**), 0.0002 (***) and <0.0001 (****)). Representative histograms are represented. *p* > 0.001 compared with negative control cells.

After the observation of antisurvival and antiproliferative effects induced by the SCFAs, we next wanted to analyze cell death by annexin V/Pi (AV/Pi) staining. Following the same rationale as in the previous assays, the analysis was performed for both CRC-derived cell lines (RKO and HCT-15) after the treatment with each SCFA alone (IC_50_ and IC_75_), with mixture II (IC_50_ and IC_75_), and with each SCFA alone at a concentration present in the IC_50_ and IC_75_ of mixture II (for values, see Table 3). We observed that all single SCFAs induce apoptotic cell death, with exposure of phosphatidylserine to the outer layer of the plasma membrane in both cell lines, according to the annexin V positive population (Figure 5a,c). Considering the mixture effects, it is possible to see that, for both cell lines, the highest concentration of mixture also induces apoptotic cell death (Figure 5A,B). While in RKO this seems to be due mostly to acetate effects (Figure 5b), in HCT-15 this is correlated with the effects induced by the butyrate and propionate at the concentration present in the mixture (Figure 5d).

### 3.5. Short-Chain Fatty Acids Increase Lactate Production in CRC Cells

**Figure 6:**
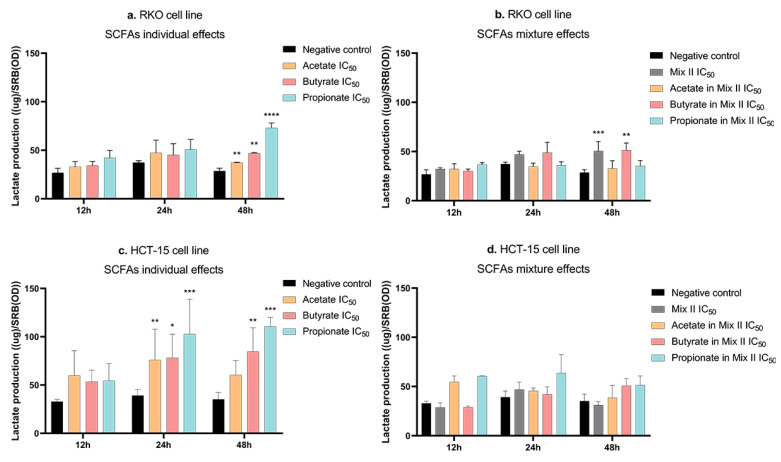
Energetic metabolism effects induced by SCFAs, individually or in mixture, in CRC cells. (**a**–**d**) Lactate-production quantification using the Lactate LO-POD enzymatic colorimetric kit from Spinreact in CRC cells treated with each SCFA, individually or in mixture, for 48 h. (**a**) RKO cells were incubated with the IC_50_ of each SCFA (81.04 mM of acetate, 10.84 mM of butyrate, 32.25 mM of propionate). (**b**) RKO cells were incubated with the IC_50_ of the mixture II, as well as the correspondent concentrations of each SCFA in that mixture (47.61 mM of Mix II containing 28.57 mM of acetate, 7.14 mM of butyrate, and 11.90 mM of propionate). (**c**) HCT-15 cells were incubated with the IC_50_ of each SCFA (89.52 mM of acetate, 4.57 mM of butyrate, 22.70 mM of propionate). (**d**) HCT-15 cells were incubated with the IC_50_ of the mixture II, as well as the correspondent concentrations of each SCFA in that mixture (13.67 mM of Mix II containing 8.20 mM of acetate, 2.05 mM of butyrate, and 3.42 mM of propionate). Cells were incubated with fresh complete medium as negative control. Data are from at least three independent experiments run in triplicate. Statistical analysis was performed using Two-Way ANOVA (*p*-value 0.0332 (*), 0.0021 (**), 0.0002 (***) and <0.0001 (****)).

Considering previous results from the group showing that acetate induces alterations in the energetic metabolism of CRC cells [21], we were interested in analyzing the remaining SCFAs and their mixture’s effects at the metabolic level [21]. With that aim, the lactate production rates were evaluated in both the RKO and HCT-15 cell lines, after 12 h, 24 h, and 48 h of treatment with SCFAs alone (IC_50_), with mixture II (IC_50_), and with SCFAs alone at a concentration present in the IC_50_ of mixture II (for values see Table 3). Data analysis of the lactate production showed that all individual SCFAs increased lactate production in RKO (after 48 h) and in HCT-15 cells (after 24 and 48 h) (Figure 6a,c). Moreover, in RKO cells, mixture II also increased lactate production. This effect was of similar magnitude to that seen with butyrate individually, at the concentration present in that mixture (after 48 h) (Figure 6b). No significant mixture effects were observed for the HCT-15 cell line at any of the timepoints tested (Figure 6d).

### 3.6. Short-Chain Fatty Acids Promote Lysosomal-Membrane Permeabilization and Induce Cytosolic Acidification in CRC Cells

**Figure 7:**
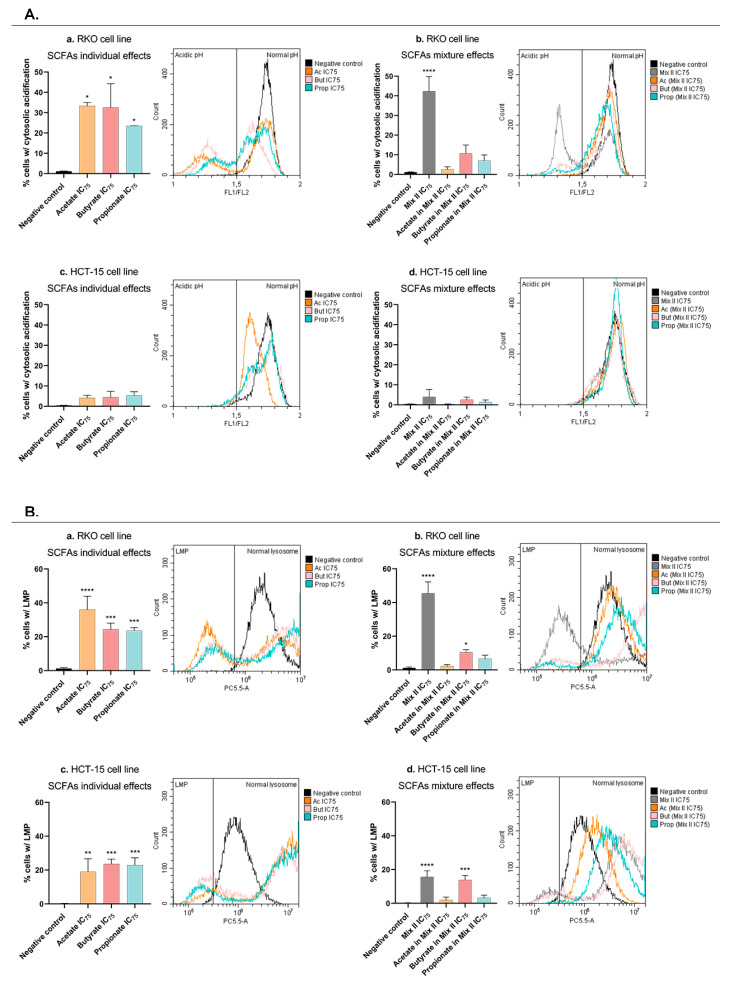
pH effects and LMP induced by SCFAs, individually or in mixture, in CRC cells. (**A**) Intracellular pH analysis by BCECF staining by flow cytometry in CRC cells treated with each SCFA, individually or in mixture, for 48 h. (**Aa**) RKO cells were incubated with the IC_75_ of each SCFA (116.60 mM of acetate, 44.16 mM of butyrate, 56.89 mM of propionate). (**Ab**) RKO cells were incubated with the IC_75_ of the mixture II, as well as the correspondent concentrations of each SCFA in that mixture (77.32 mM of Mix II containing 46.39 mM of acetate, 11.60 mM of butyrate, and 19.33 mM of propionate). (**Ac**) HCT-15 cells were incubated with the IC_75_ of each SCFA (161.55 mM of acetate, 14.56 mM of butyrate, 50.70 mM of propionate). (**Ad**) HCT-15 cells were incubated with the IC_75_ of the mixture II, as well as the correspondent concentrations of each SCFA in that mixture (39.97 mM of Mix II containing 23.98 mM of acetate, 6.00 mM of butyrate, and 9.99 mM of propionate). Data are from at least three independent experiments. *p* > 0.001 compared with negative control cells. (**B**) LMP analysis by AO staining by flow cytometry in CRC cells treated with each SCFA, individually or in mixture, for 48 h. (**Ba**) RKO cells were incubated with the IC_75_ of each SCFA (116.60 mM of acetate, 44.16 mM of butyrate, 56.89 mM of propionate). (**Bb**) RKO cells were incubated with the IC_75_ of the mixture II, as well as the correspondent concentrations of each SCFA in that mixture (77.32 mM of Mix II containing 46.39 mM of acetate, 11.60 mM of butyrate, and 19.33 mM of propionate). (**Bc**) HCT-15 cells were incubated with the IC_75_ of each SCFA (161.55 mM of acetate, 14.56 mM of butyrate, 50.70 mM of propionate). (**Bd**) HCT-15 cells were incubated with the IC_75_ of the mixture II, as well as the correspondent concentrations of each SCFA in that mixture (39.97 mM of Mix II containing 23.98 mM of acetate, 6.00 mM of butyrate, and 9.99 mM of propionate). Data are from at least three independent experiments. Statistical analysis was performed using One-Way ANOVA (*p*-value 0.0332 (*), 0.0021 (**), 0.0002 (***) and <0.0001 (****)).

Knowing that acetate-induced apoptosis is related to lysosomal-membrane permeabilization (LMP) [23] the next step of the work was to evaluate if butyrate, propionate, or the mixture-induced apoptotic effects are also associated with LMP and with a consequent acidification of CRC cells’ cytosol. In order to evaluate LMP, acridine orange (AO) staining was performed in RKO and HCT-15 cells after treatment with each SCFA alone (IC_75_), with mixture II (IC_75_), and with each SCFA alone at a concentration present in the IC_75_ of mixture II (for values, see Table 3). Flow-cytometry analysis demonstrated that, in both cell lines, acetate, butyrate, and propionate, individually, significantly induce LMP, when in comparison with the negative control cells (Figure 7Aa,Bc). Similarly, to each individual SCFA, mixture II also promotes LMP, and in the case of the HCT-15 cells, this result seems to be followed by the effects of the butyrate alone (Figure 7Ac,Ad).

Considering the intracellular pH, the same experimental design was followed, but instead of AO, the RKO and HCT-15 cells were stained with a BCECF probe, a well-established fluorescent probe that enables the ratiometric monitoring of cellular pH. A cytosolic acidification was detected in RKO cells treated with each individual SCFA, as well as with mixture II (Figure 7Ba,Bc). For the HCT-15 cell line, no significant differences were observed with any treatment (Figure 7Bb,Bd).

## 4. Discussion

CRC continues to be the main cause of cancer-related deaths worldwide [1,43]. The classical chemotherapy with 5-fluorouracil constitutes a clinical problem, due to the high resistance, resulting in response rates of less than 10% [43]. It is known that dietary habits have implications on the colon microbiota and play a role in basic biological processes, such as the production of several metabolites, namely SCFAs (acetate, butyrate, and propionate) [44,45]. In addition, SCFAs are known to be decreased in CRC patients due to a microbiota dysbiosis. This work arises from the need to find a new strategy based on the modulation of the colon microbiota to challenge CRC treatment.

The scientific community has been interested in exploring the role that each SCFA individually plays in CRC carcinogenic processes [21,22,23,46]. As such, significant advances have been made in the understanding of the individual effects of SCFAs. For example, butyrate has been shown to inhibit cell differentiation, induce apoptotic cell death, and block cell proliferation by promoting cell-cycle arrest [13,14,15,16,17,18]. Similarly, reports have demonstrated that propionate treatment induces apoptosis, necrosis, or autophagy and leads to an anti-inflammatory patterns, also promoting some histone acetylation [18,19,20]. Our research group has also demonstrated that acetate leads not only to the inhibition of proliferation but also to the induction of cell death by apoptosis, with promotion of LMP and release of cathepsin D [18,21,22,23]. Although the role of individual SCFAs has been widely studied, colon cells are exposed to these three compounds in combination, and, in this regard, a study defining the effects of SCFA mixtures in the biological hallmarks of CRC, to the best of our knowledge, was never conducted.

In order to fully understand the combined effects of SCFAs, we first aimed at conducting proof-of-concept experiments to ensure that we could quantify the mixture effects in our test systems. The study of this SCFA mixture’s effect was possible by applying a well-established mathematical model for the calculation of expected additive mixture effects, the CA model. The use of the CA model requires an exhaustive characterization of the concentration–effect relationships of each individual mixture component [28,29]. The SRB assay was performed to meet these requirements, allowing reproducible results and complete curves that span a wide concentration–effect range for each SCFA. From all the individual dose-response curves obtained and IC_50_ calculated, butyrate was shown to induce more potent responses than acetate or propionate, both in CRC cells (RKO and HCT-15) and in nonneoplastic cells (NCM460). The selectivity index revealed that each one of the three SCFAs selectively affect CRC cells, since the doses required for inhibiting 50% of cell growth of the cancer cell lines are lower than the doses required for NCM460 cells. Our results demonstrated that all SCFAs were selective for CRC cells, butyrate being the more potent one towards CRC.

Concerning the comparisons between the predicted and experimentally observed effects of the SCFAs mixtures, it was clear that the CA model is the appropriate tool for the assessment of additive joint effects of mixtures of SCFAs in an “in vitro” model of CRC. The overlap between the predicted data and the best-fit regression model for the SRB results showed good conformity in RKO cells, demonstrating, for the first time, that the three tested compounds act in an additive fashion to produce the overall mixture effect. This means that the contribution of each SCFA to the overall mixture effect should be proportional to its concentration, even at concentrations that individually have an undetectable impact. This same pattern is not observed for NCM460 normal colon cells. For mixture I, the SCFA seems to have an additive effect in the cancer cell lines and a synergistic effect in the normal cell line. Interestingly, for mixture II, wherein SCFAs are combined at realistic ratios, synergism is also observed in HCT-15. This is promising as it seems to indicate that, when combined at concentrations present in the colon, SCFAs are able to interact with each other to reduce the growth of cancer cells in a more significant way than would be expected based solely on their individual effects.

We showed that all SCFAs induce LMP in CRC-derived cells, indicating that one of the intracellular targets of these three SCFAs should be the lysosome. This organelle is extremely important in cancer development and progression, contributing to cancer-cell proliferation, invasion and metastasis, angiogenesis, and also being an important destruction site for immune-checkpoint molecules [47]. In comparison with noncancerous cells, it has been shown that the activity of multiple enzymes in lysosomes is significantly increased in many cancer tissues [47,48]. Considering the lysosome contributions for the carcinogenic process and the differences in activity and distribution within the cancerous and noncancerous cells, it is plausible to hypothesize that the different toxicologic mixture behavior observed in the RKO and HCT-15 cells when compared with NCM460 cells could be explained based on the lysosome targeting.

Once we understood how the three SCFAs acted in combination, we aimed to evaluate how this mixture affects several biological processes that occur during colorectal carcinogenesis, and also to compare them with the effects induced by each SCFA individually. SRB results showed decrease in cell growth induced by SCFAs, alone or in mixture. In this regard, the question arose as to whether this decrease was due to the inhibition of cell proliferation and/or increased cell death. Our data demonstrated that the decrease in cellular growth induced by the SCFAs (both alone and sin mixture) could be explained in two phases. Firstly, they inhibited cell proliferation, verified through cell-cycle arrest after 24 h of treatment. Secondly, they increased cell death by apoptosis, verified 48 h after treatment. It is also important to notice that, in all the experiments, when we compare the SCFA mixture effects with the effects of each SCFA individually (at the concentrations present in the mixture), a tendency for the mixture to follow the butyrate behavior is very representative. These interesting results seem to indicate that butyrate has the ability to “command” the mixture behavior, which must be related to its significantly higher potency in relation to the other SCFAs, seeing as it is present at the lowest percentage in the mixture (molecular ratio of mixture II—60 acetate:15 butyrate:25 propionate). Previous reports from our group [21], showing that acetate treatment per se induces changes in the glycolytic metabolic profile of CRC cells, raised a similar question with regards to the remaining two SCFAs and the mixture [21]. Similar to what happens with acetate, the treatment with butyrate, propionate, and the mixture induced an increase in the production of lactate by CRC cells. The Warburg effect is a metabolic adaptation that happens when, even in the presence of sufficient amounts of oxygen, cancer cells use glycolysis instead of the oxidative phosphorylation to satisfy their high energy needs and sustain a high proliferative rate [49]. This metabolic process is reflected by a high level of lactate production in cancer cells that needs to be exported to prevent intracellular acidification and, consequently, cell death. Our results appear to indicate that SCFAs promote energy production through the potentiation of glycolysis, thus producing more lactate. Given that acetate-induced apoptosis in CRC cells has been shown to involve LMP [23], we considered it important to finish this phenotypic characterization with the study of the effects of the three SCFAs, alone and combined, on LMP and the modulation of the cytosolic pH. The results were clear showing that when CRC cells are treated with a considerable dose of acetate, butyrate, propionate, or mixture, permeabilization of the membrane of the lysosome occurs and intracellular pH decreases. Based on all these results, we can conclude that, similarly to what we had already described for acetate, the mechanism of action of all SCFAs, individually or in a mixture, is associated with LMP and consequent cytosol acidification, which leads to apoptotic cell death [22,23]. Considering the current literature describing enhanced lysosomal activity in cancer cells, when compared to normal cells, it is plausible that this lysosome targeting explains the high selectivity to CRC cells found for all SCFAs [47,50,51]. This has a high therapeutic potential, since a strategy based on increasing SCFA levels could constitute an alternative approach that preferentially targets CRC cells, with reduced effects on normal colon cells.

One possible strategy for increasing SCFAs levels in CRC patients could be the modulation of the intestinal microbiota composition towards more favorable species for SCFA production. CRC patients are characterized by increased levels of *Bacteroidetes*, *Firmicutes*, and *Fusobacteria* and decreased *Proteobacteria* in the gut [18]. In this regard, counteracting this tendency by increasing SCFA-producing bacteria, namely *Propionibacteria*, could constitute an important alternative. Specific dietary habits, such as the consumption of dairy products, are described among the strongest modulators of gut microbiota composition and function [52]. Bioactive candidates, such as probiotics, dietary fibers, and nondigested proteins can intervene in the proliferation and/or stimulation of the metabolic activity of bacterial populations contributing to the modulation of SCFA levels [52]. Our group has already investigated the potential of *Propionibacterium freudenreichii* for use as a probiotic and revealed that its ability to produce high amounts of acetate and propionate contributes to the inhibition of CRC cell proliferation and induction of cell-cycle arrest. This suggests that the use of *P. freudenreichii* as a probiotic could constitute a therapeutic adjuvant strategy for CRC [46].

**Figure 8:**
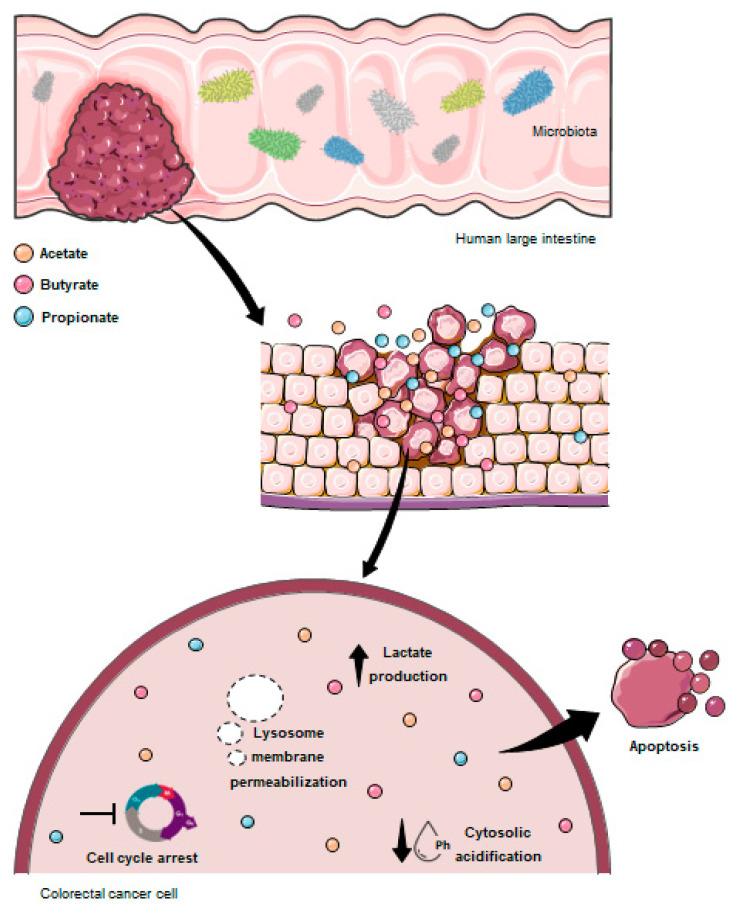
Schematic representation of the SCFA effects on CRC cells. Acetate (orange circle), butyrate (pink circle), and propionate (blue circle) derived from the intestinal microbiota are selective for CRC cells. Once inside the CRC cells, SCFAs interfere with several biological effects, namely inhibiting cell survival, inducing cell-cycle arrest and apoptosis, promoting LMP with a consequent cytosolic acidification, and, also, increasing lactate production.

In summary, here we showed that the combination of all three SCFAs have potential therapeutic effects against CRC. To our knowledge, this was the first “in vitro” experimental work studying SCFA mixture behavior using a mathematical model and showing the antitumoral effects of acetate, butyrate, and propionate combined at the physiological proportions founded in the human colon. We showed, for the first time, that SCFAs in physiological mixture are involved in the regulation of several biological processes, namely cell survival, proliferation, apoptosis, energetic metabolism, cytosolic pH, and LMP (Figure 8). These findings may contribute to the development of new alternatives for CRC therapeutics based on the increase in the SCFA levels through the modulation of the colon microbiota composition.

## Figures and Tables

**Table 1:** Parameters for the SCFAs agents in the SRB assay.

Cell Line	Compound	Estimated Parameters for the Best-Fit Regression Model of Each Individual Agent					Relative Proportion (%)	
		Regression Model	θ1	θ2	θmin	θmax	Mix I	Mix II
RKO	Acetate	Weibull	−6.95 × 10^0^	2.32 × 10^1^	0.00	1.00	65.29	60.00
	Butyrate	Weibull	−1.21 × 10^0^	5.12 × 10^0^	0.00	1.00	8.73	15.00
	Propionate	Weibull	−3.05 × 10^0^	1.33 × 10^1^	0.00	1.00	25.98	25.00
HCT-15	Acetate	Logit	−4.21 × 10^0^	1.21 × 10^1^	0.00	1.00	76.65	60.00
	Butyrate	Weibull	8.70 × 10^2^	4.05 × 10^0^	0.00	1.00	3.91	15.00
	Propionate	Weibull	−1.13 × 10^0^	7.18 × 10^1^	0.00	1.00	19.44	25.00
NCM460	Acetate	Weibull	−8.05 × 10^0^	2.40 × 10^1^	0.00	1.00	60.31	60.00
	Butyrate	Weibull	−1.85 × 10^0^	6.51 × 10^0^	0.00	1.00	13.35	15.00
	Propionate	Logit	−4.00 × 10^0^	1.26 × 10^1^	0.00	1.00	26.33	25.00

**Table 2:** IC_50_ and selectivity index determination. IC_50_ values were estimated from the dose/growth response curves of SCFAs determined by SRB assay after 48 h of treatment in normal (NCM460) and CRC (HCT-15, RKO) cells. Values derived from at least three independent experiments. The selectivity index was calculated through the division of the IC_50_ values obtained for each compound in the two CRC-derived cells, RKO and HCT-15, by the IC_50_ value obtained for the same compound in normal colon-derived cells, NCM460.

Compound	IC_50_ (mM)			Selectivity Index	
	RKO	HCT-15	NCM460	RKO	HCT-15
Acetate	81.04 ± 2.24	89.52 ± 3.31	117.30 ± 2.99	1.45	1.31
Butyrate	10.84 ± 0.79	4.57 ± 0.30	25.96 ± 1.80	2.39	5.68
Propionate	32.25 ± 1.14	22.70 ± 1.59	51.21 ± 3.42	1.59	2.26

**Table 3:** Concentrations of SCFAs used for the comparison between the individual and combined effects.

Cell lines	Dose	Mixture II (mM)	Acetate in Mixture II (mM)	Butyrate in Mixture II (mM)	Propionate in Mixture II (mM)
RKO	IC_75_	77.32	46.39	11.60	19.33
	IC_50_	47.61	28.57	7.14	11.90
	IC_25_	28.51	11.17	4.28	7.13
HCT-15	IC_75_	39.97	23.98	6.00	9.99
	IC_50_	13.67	8.20	2.05	3.42
	IC_25_	4.79	2.87	0.72	1.20

## Data Availability

Not applicable.
